# Factors predicting outcome after pulmonary endarterectomy

**DOI:** 10.1371/journal.pone.0198198

**Published:** 2018-06-21

**Authors:** Cécile Tromeur, Xavier Jaïs, Olaf Mercier, Francis Couturaud, David Montani, Laurent Savale, Mitja Jevnikar, Jason Weatherald, Olivier Sitbon, Florence Parent, Dominique Fabre, Sacha Mussot, Philippe Dartevelle, Marc Humbert, Gérald Simonneau, Elie Fadel

**Affiliations:** 1 European Brittany University, Brest, France; 2 Department of Internal Medicine and Chest Diseases, University Hospital Centre La Cavale Blanche, Brest, France; 3 Groupe d’Etude de la Thrombose de Bretagne Occidentale (GETBO), EA 3878, CIC INSERM 1412, Brest, France; 4 Univ Paris-Sud, Faculté de Médecine, Université Paris-Saclay, Le Kremlin-Bicêtre, France; 5 AP-HP, Service de Pneumologie, Centre de Référence de l’Hypertension Pulmonaire, Hôpital Bicêtre, Le Kremlin-Bicêtre, France; 6 INSERM UMR_S 999, Hôpital Marie Lannelongue, Le Plessis Robinson, France; 7 Service de Chirurgie Thoracique et Vasculaire et de Transplantation Cardiopulmonaire, Hôpital Marie Lannelongue, Le Plessis Robinson, France; 8 Department of Medicine, Division of Respirology, University of Calgary, Calgary, Alberta, Canada; Stanford University, UNITED STATES

## Abstract

**Objective:**

Few studies have reported predictive factors of outcome after pulmonary endarterectomy (PEA) in chronic thromboembolic pulmonary hypertension. The purpose of this study was to determine factors influencing mortality and predictors of hemodynamic improvement after PEA.

**Methods:**

A total of 383 consecutive patients who underwent PEA between January 2005 and December 2009 were retrospectively reviewed. Among them, 150 were fully reevaluated 7.5±1 months after PEA by NYHA class, 6–minute walk distance (6MWD), percentage of predicted carbon monoxide transfer factor (TL_CO_) and right heart catheterisation.

**Results:**

Mortality rates at 1 month, 1 year and 3 years were 2.8%, 6.9% and 7.5%, respectively. Preoperative pulmonary vascular resistance (PVR) independently predicted 1-month, 1- and 3-year mortality and age predicted mortality at 1 year and 3 years. Significant improvement in NYHA class and 6MWD were observed and PVR decreased from 773±353 to 307±221 dyn.sec.cm^-5^ (p<0.001). In 96 patients (64%), PVR decreased by at least 50% and/or was reduced to lower than 250 dyn.sec.cm^-5^. Preoperative cardiac output (CO) and TL_CO_ predicted hemodynamic improvement.

**Conclusion:**

PEA is associated with an excellent long-term survival and a marked improvement in clinical status and hemodynamics. Some preoperative factors including PVR, CO and TL_CO_ can predict postoperative outcomes.

## Introduction

Chronic thromboembolic pulmonary hypertension (CTEPH) is a rare complication of venous thromboembolism and is associated with an important morbidity and mortality. CTEPH results from obstruction of the pulmonary arterial bed by organized thrombus after acute or recurrent pulmonary emboli. Despite the advent of medical therapies [1] and the emergence of pulmonary angioplasty for CTEPH [2], the best-established treatment remains pulmonary endarterectomy (PEA), which is usually performed in expert surgical centers [3]. Eligibility criteria for surgery are determined by a multidisciplinary panel of physicians, surgeons and radiologists and are based on the amount of surgically accessible lesions assessed by imaging, the presence of comorbidities and the degree of hemodynamic impairment in symptomatic patients [3]. When successful, PEA markedly improves pulmonary hemodynamics, symptoms and functional status. However, several studies have demonstrated that some patients had persistent pulmonary hypertension (PH) after the procedure [4–7] Persistent PH after surgery represents the most important cause of postoperative morbidity and mortality but there is no consensus on its definition. Some authors used mean pulmonary artery pressure (mPAP) thresholds of 25–30 mmHg [5, 7] whereas others used pulmonary vascular resistance (PVR) thresholds of 500–550 dyn.sec.cm^-5^ [4, 6, 8], although mPAP≥25mmHg is the accepted definition of PH (3).

In other studies, it has been shown that high preoperative pulmonary vascular resistance (PVR), thought to reflect the degree of distal arteriopathy, is associated with increased PEA-related mortality among operable patients [6, 7, 9–11]. There are also several groups reporting the long-term outcome after PEA [4, 8, 12–15] but there is little data regarding the factors that influence the hemodynamic and functional improvement [11, 16, 17].

In a cohort of consecutive CTEPH patients who underwent PEA, we aimed to identify: (1) factors affecting short and long-term survival; (2) predictive factors of hemodynamic and/or functional status improvement.

## Patients and methods

This retrospective study complied with the Declaration of Helsinki. Although French law does not require ethics committee approval or informed consent for retrospective data collection, the data was anonymised and complied with the requirements of the Commission Nationale Informatique et Liberté, the organisation dedicated to privacy, information technology, and civil rights in France. The committee approved the methods used to collect and analyse data on May 24, 2003 (approval number 842063).

### Population

We retrospectively reviewed the charts of all newly diagnosed consecutive patients with CTEPH who underwent PEA between 2005 and 2009 in the French reference centre for pulmonary hypertension (PH). A diagnosis of precapillary PH was established according to current guidelines (mean pulmonary artery pressure (mPAP) ≥25mmHg and pulmonary artery wedge pressure (PAWP) ≤15mmHg measured by right heart catheterization) [3]. CTEPH was confirmed as the cause of pulmonary hypertension in the presence of mismatched perfusion defects on radionuclide ventilation/perfusion lung scan and typical lesions of CTEPH on computed tomography pulmonary angiogram and/or conventional pulmonary angiography. Patients were required to have received at least 3 months of adequate anticoagulation therapy before PEA.

Patients were not included in the analysis if the preoperative PVR was below 250 dyn.sec.cm^-5^, or in the absence of either a preoperative hemodynamic assessment or postoperative follow-up.

### Data collection and classification

Data were collected from the French PH registry or from routinely performed assessments in clinical practice. Predefined clinical, functional, hemodynamic and therapeutic data were collected using a standardized case report form. The period of observation was from the date of PEA until time of death or date of last known contact.

Data were categorized into five sets of parameters: (1) clinical (age, gender, smoking, history of acute pulmonary embolism, associated medical conditions); (2) therapeutic (delay between first assessment and PEA, use of pulmonary arterial hypertension (PAH) targeted treatment before PEA, duration of treatment before PEA); (3) functional (New York Heart Association [NYHA] functional class and 6-minute walk distance [6MWD]); (4) spirometric (TL_CO_ expressed as % of the predicted value); and (5) hemodynamic (PVR expressed in dyn.sec.cm^-5^, mPAP expressed in mmHg, cardiac output (CO) expressed in L.min^-1^).

### Outcomes evaluation

The primary outcome was short-term (within 1 month after surgery, i.e.; the early post-operative period) and long-term mortality (at 1 year and 3 years) after PEA.

Secondary outcomes were:

1. normalization of hemodynamics, defined by postoperative PVR <250 dyn.sec.cm^-5^ at first assessment after PEA;
2. clinically meaningful improvement in hemodynamics defined, as it has been suggested in previous publications [9, 18], by a postoperative decrease in PVR by at least 50% and to an absolute value of less than 500 dyn.sec.cm^-5^ at first assessment after PEA. The threshold of 500 dyn.sec.cm^-5^ was chosen because it is strongly correlated with mortality in several studies [4, 6, 8]. Patients with preoperative PVR below 500dyn.sec.cm^-5^ were not included in this analysis;
3. improvement in NYHA by at least one functional class at first evaluation after PEA.

### Statistical analysis

Continuous variables were expressed as mean (± standard deviation [17]) or median depending on normality of the distribution; 95% confidence intervals (95% CI) were computed for proportions according to the binomial distribution. For the comparison of pre- and postoperative data samples a paired Student’s *t*-test was used for continuous variables and a Wilcoxon test for the improvement of NYHA classification. Univariate analysis was conducted in four steps: (1) an association between each predefined variable and endpoints was tested using univariable logistical regression and only variables with a p-value <0.2 were considered in further analysis; (2) correlations between variables were systematically searched within each and between sets of variables (in case of significant correlation, the most statistically significant variable was considered); (3) for continuous variables that were statistically significant, the discriminant power was determined by calculating the area under the curve (AUC) on receiver operating characteristic (ROC) curve analysis; and (4) interactions between variables (within and between sets of variables) were also estimated and considered for a p-values <0.1. Multivariate analysis was performed using a multivariable logistic regression. In the model, only variables with a p-value <0.2, which were not correlated and had no significant interaction (p-value >0.1), were included. Age and FEV1/FVC were also systematically included in the model. In this multivariate model, predictors were considered as statistically significant for p-value of less than 0.05. Survival curves were derived by Kaplan-Meier method. Statistical analyses were performed using SPSS software (version 19.0; SPSS, Inc.; Chicago, Illinois).

## Results

### Study population

**Fig 1:**
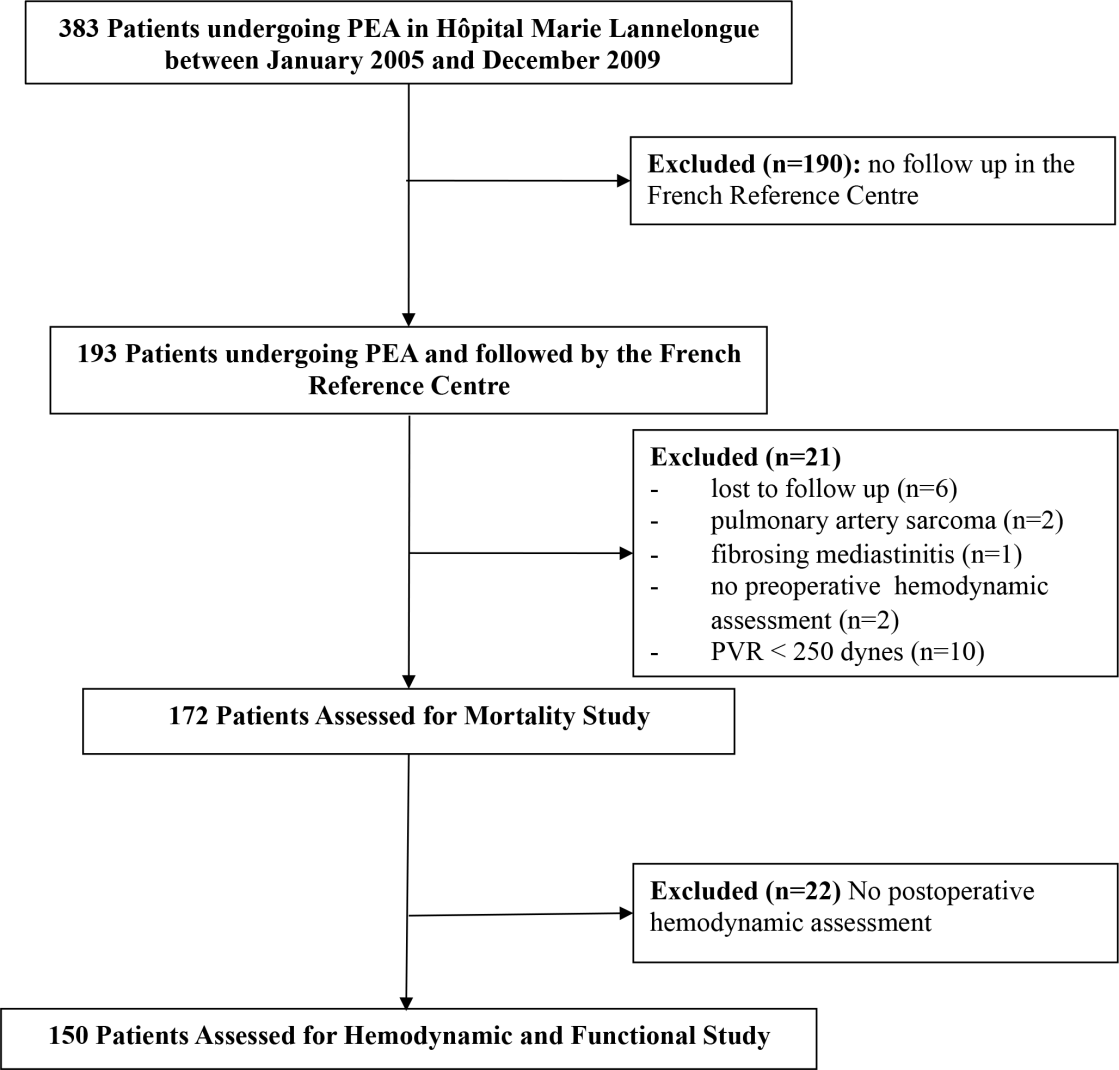
Flow chart of the study population.

Between January 2005 and December 2009, 383 patients underwent PEA. Among them, 193 were followed up after surgery at the French reference for PH. Twenty-one patients were excluded from the study for the following reasons: loss of follow-up (n = 6), final diagnosis of sarcoma (n = 2), final diagnosis of fibrosing mediastinitis (n = 1), no preoperative hemodynamic assessment (n = 2) and PEA performed in patients with PVR <250 dyn.sec.cm^-5^ (n = 10). Therefore, the primary objective (mortality) was analysed in 172 patients. As full postoperative hemodynamics were not assessed in 22 of these patients, the secondary endpoints were evaluated in 150 patients (**Fig 1**). The mean age of the patients was 60±14 years and 51% were female. A history of acute venous thromboembolism was reported in 114 patients (66%). At the time of surgery, 26 patients (15%) had medical conditions increasing the risk of CTEPH and including antiphospholipid syndrome (n = 6), splenectomy (n = 6), myeloproliferative disorder (n = 12), ventriculoatrial shunt (n = 4), implanted central venous access port (n = 3) and cardiac pacemaker (n = 4). Pre-PEA, 19 patients (11%) were receiving at least one PAH targeted treatment. The time between diagnosis of CTEPH and PEA was significantly longer in these patients than in patients who did not receive PAH targeted therapies before surgery (8.1±5 versus 1.2±1.9 months; p<0.001). At the time of pre-surgery evaluation, patients receiving bridging therapy had higher mPAP (55±12 versus 46±10 mmHg; p<0.001) but the level of PVR did not differ between the two groups (858±338 versus 754±341 dyn.sec.cm^-5^; p = 0.14). The preoperative patient characteristics are summarized in **Table 1**.

**Table 1:** Baseline patient characteristics.

Characteristics	Study Population for Mortality Assessment	Study Population for Haemodynamic Assessment
	n = 172*	n = 150**
**Clinical Characteristics**		
Age—years, mean ± SD	60 ± 14	60 ± 14
Gender		
Female–n	88	81
Male–n	84	69
BMI, mean ± SD	27 ± 6.3	27 ± 6.5
History of smoking–n (%)	12 (7)	12 (8)
History of VTE–n (%)	114 (66)	100 (66)
Associated Medical conditions–n (%)	26 (15)	17 (11)
**Functional Characteristics**		
NYHA functional class–n		
I	1	1
II	28	25
III	111	99
IV	32	25
6MWD (meters), mean ± SD	293 ±168	310 ±159
**Hemodynamic Characteristics**		
mPAP (mmHg), mean ± SD	47 ± 11	48 ± 11
CO (L.min^-1^), mean ± SD	4.53 ± 1.37	4.53 ± 1.35
PVR (dynes.s.cm^-5^), mean ± SD	768 ± 342	773 ± 353
**Spirometric Characteristics**		
FEV1 (% of predicted value), mean ± SD	84 ± 20	84 ± 20
FVC (% of predicted value), mean ± SD	83 ± 18	83 ± 18
TL_CO_ (% of predicted value), mean ± SD	63 ± 15	63 ± 15
FEV1/FVC (%), mean ± SD	76 ± 9	76 ± 9
PaO2 (mmHg), mean ± SD	64 ± 12	64 ± 13
**Therapeutic Characteristics**		
Initiation of PAH targeted treatment before PEA–n (%)	19 (11)	18 (12)
Duration of PAH targeted treatment before PEA (months), mean ± SD	8.1 ± 5	8 ± 6

*Abbreviations*: BMI, body mass index; VTE, venous thromboembolism; NYHA, New York Heart Association; 6MWD, 6-min walk distance; mPAP, mean pulmonary arterial pressure; CO, cardiac output; PVR, pulmonary vascular resistance; FEV1, force expiratory volume in one second; FVC, forced vital capacity; TL_CO_, transfer coefficient of the lung for carbon monoxide; PaO2, partial pressure of oxygen in arterial blood; PAH, pulmonary arterial hypertension; PEA, pulmonary endarterectomy.

*172 patients assessed for mortality.

**150 patients assessed for predictive factors of hemodynamic normalization and functional or hemodynamic improvement.

### Primary outcome

**Fig 2:**
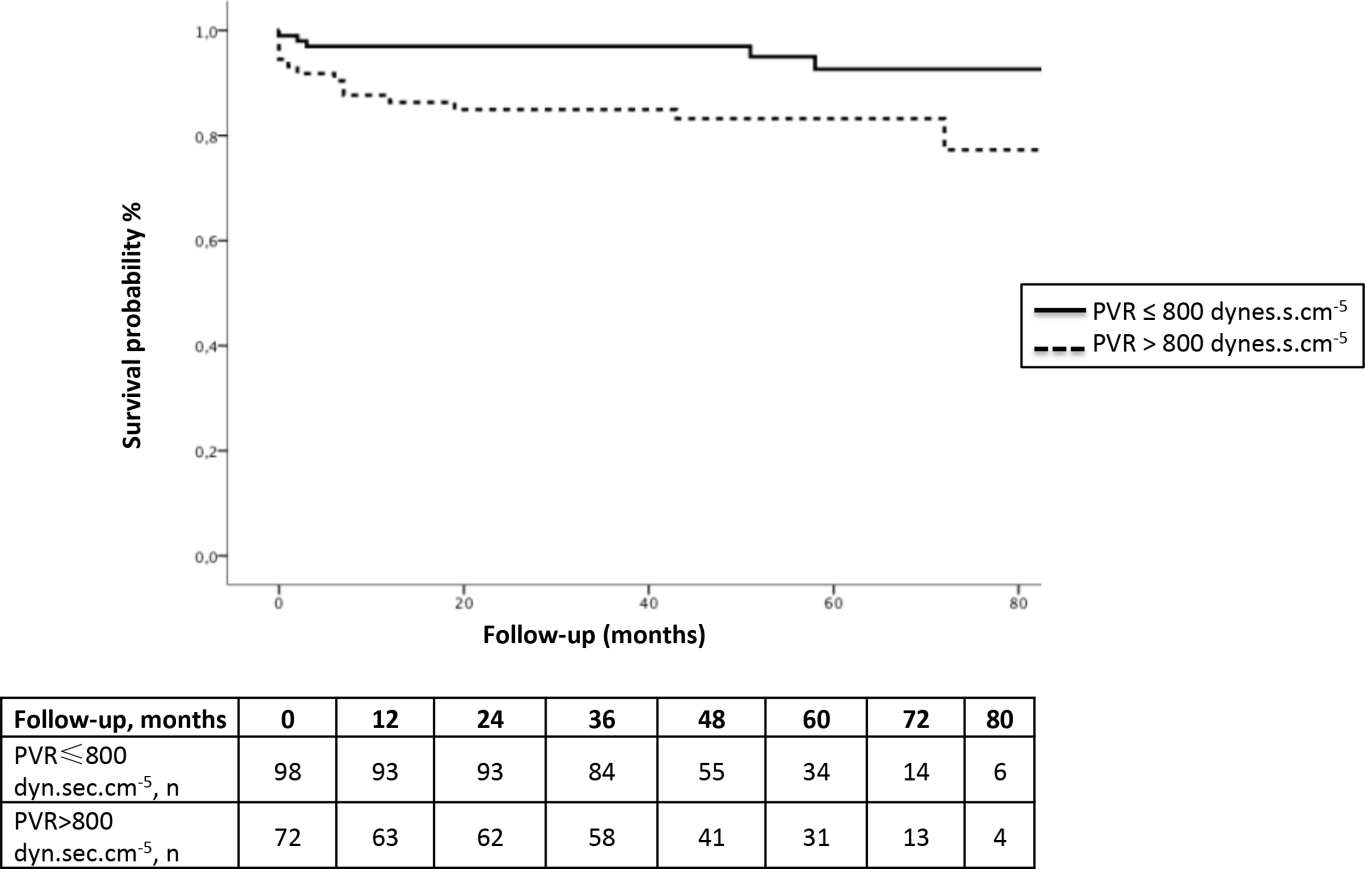
Survival curves according to the optimal cut-off value derived by ROC analysis for preoperative pulmonary vascular resistance (PVR).

Postoperative mortality rates at 1 month, 1 year and 3 years were 2.9%, 6.9% and 7.5%, respectively. Early mortality was mainly related to right ventricular failure. The causes of death are detailed in S1 Appendix. In multivariate analysis, preoperative PVR was a predictive factor of mortality at 1 month (odd ratio (OR, per 100dyn.sec.cm^-5^) 1.329, 95% confidence interval (CI), 1.035 to 1.706, p = 0.026), 1 year (OR, per 100dyn.sec.cm^-5^ 1.252, 95% CI, 1.024 to 1.529, p = 0.028) and 3 years (OR, per 100dyn.sec.cm^-5^ 1.245, 95% CI, 1.013 to 1.529, p = 0.037). Age also predicted 1- and 3-year mortality. There was a trend towards a deleterious effect of the use of PAH targeted therapies before PEA (duration of use before surgery, per month) on 1-month mortality (p = 0.053). The results of the univariate and multivariate analyses are shown in **Table 2**. No other baseline factors were predictors of mortality in univariate and multivariate analysis. Survival according to the optimal threshold of PVR determined by ROC analysis is represented in **Fig 2** (AUC = 0.72, standard error 0.06, p = 0.008, sensitivity 77%, specificity 60%).

**Table 2:** Predictive factors of mortality.

Predictors		Mortality at 1 month				Mortality at 1 year				Mortality at 3 years			
		Univariable		Multivariable		Univariable		Multivariable		Univariable		Multivariable	
		**OR** **(95%CI)**	**P**	**OR (95%CI)**	**P**	**OR** **(95%CI)**	**P**	**OR (95%CI)**	**P**	**OR** **(95%CI)**	**P**	**OR (95%CI)**	**P**
Duration of PAH targeted treatment before PEA		1.183 (1.043–1.342)	**0.009**	1.47 (1.00–2.17)	**0.053**	1.160 (1.041–1.292)	**0.007**	1.087 (0.590–2.002)	**0.79**	1.151 (1.034–1.280)	**0.01**	1.088 (0.586–2. 019)	**0.79**
PVR*(per 100dyn.sec.cm^5^)		1.231 (0.997–1.521)	**0.054**	1.329 (1.035–1.706)	**0.026**	1.201 (1.034–1.394)	**0.016**	1.252 (1.024–1.529)	**0.028**	1.205 (1.042–1.393)	**0.012**	1.245 (1.013–1.529)	**0.037**
**Age**		1.015 (0.949–1.085)	**0.67**			1.069 (1.006–1.137)	**0.032**	1.084 (1.005–1.168)	0.036	1.074 (1.011–1.141)	**0.02**	1.087 (1.010–1.170)	**0.026**

*Abbreviations*: OR, Odds Ratio; CI, Confidence Interval; PAH, pulmonary arterial hypertension; PEA, pulmonary endarterectomy; PVR, pulmonary vascular resistance.

* There is a strong correlation between PVR and cardiac Output (CO) (r = 0.7, p <0.001). PVR and CO influenced mortality similarly.

### Secondary outcomes

The mean delay between PEA and first postoperative assessment was 7.5±1 months (range 0–60 months). Significant improvements in NYHA class (83% of patients were in NYHA III or IV before surgery and 75% were in NYHA I or II at first postoperative assessment; p<0.001) and 6MWD (310±159 versus 399±146 m; p<0.001) were observed. Mean PAP decreased from 48±11 mmHg to 28±10 mmHg, PVR declined from 773±353 to 307±221 dyn.sec.cm^-5^ and CO increased from 4.53±1.35 L.min^-1^ to 5.50±1.41 L.min^-1^ (p<0.001) (**Table 3**).

**Table 3:** Comparison of pre- and postoperative clinical and hemodynamic parameters.

Characteristics	Preoperative Assessment	Postoperative Assessment	P
**NYHA classes (n)**			
**I**	**1**	**53**	< 0.001*
**II**	**25**	**60**	
**III**	**99**	**23**	
**IV**	25	3	
**6MWD (meters), mean ± SD**	**310 ± 159**	**399 ± 146**	< 0.001**
**Hemodynamic assessment** **mPAP (mmHg), mean ± SD**	**48 ± 11**	**28 ± 10**	< 0.001**
**PVR (dynes.s.cm^-5^), mean ± SD**	**773 ± 353**	**307 ± 221**	< 0.001**
**CO (L.min^-1^), mean ± SD**	**4.53 ± 1.35**	**5.50 ± 1.41**	< 0.001**

*Abbreviations*: NYHA, New York Heart Association; 6MWD, 6-min walk distance; mPAP, mean pulmonary arterial pressure; CO, cardiac output; PVR, pulmonary vascular resistance.

* Wilcoxon test.

** Paired Student’s *t*-test.

### Normalization of pulmonary hemodynamics

Normalization of hemodynamics, defined by postoperative PVR <250 dyn.sec.cm^-5^, was observed in 71 out of the 150 patients (47%). Multivariate analysis revealed that preoperative CO was the main predictive factor of normalization of hemodynamics (**Table 4**).

**Table 4:** Predictive factors of hemodynamic normalization*.

	Univariable Analysis		Multivariable Analysis	
	OR (95%CI)	p	OR (95%CI)	p
**Clinical Characteristics**				
**Age—years**	0.98 (0.95–0.999)	0.042	**0.99 (0.96–1.02)**	**0.052**
**Functional Characteristics**				
**NYHA functional class**	0.41 (0.23–0.75)	0.004	**0.50 (0.25–1.00)**	**0.051**
**6MWD (meters)**	1.003 (1.001–1.005)	0.015		
**Hemodynamic Characteristics**				
**CO (L.min^-1^)**	1.42 (1.10–1.84)	0.007	**1.44 (1.04–2.00)**	**0.028**
**PVR (dynes.s.cm^-5^)**	0.999 (0.998–1.000)	0.03		
**Spirometric Characteristics**				
**TL_CO_ (% of predicted value)**	1.027 (1.003–1.053)	0.03	**1.015 (0.989–1.042)**	**0.27**
**FEV1/FVC (%)**	1.026 (0.99–1.065)	0.18	**1.032 (0.987–1.079)**	**0.17**
**Therapeutic Characteristics**				
**Introduction of PAH targeted treatment before PEA**	1.19 (0.5–0.68)	**0.011**	**0.37 (0.00–35.00)**	**0.67**

*Abbreviations*: OR, Odds Ratio; CI, Confidence Interval; NYHA, New York Heart Association; 6MWD, 6-min walk distance; CO, cardiac output; PVR, pulmonary vascular resistances; FEV1, forced expiratory volume in one second; FVC, forced vital capacity; TL_CO_, transfer coefficient of the lung for carbon monoxide; PAH, pulmonary arterial hypertension; PEA, pulmonary endarterectomy.

*Hemodynamic normalization was defined by PVR <250 dynes.s.cm^-5^ and occurred in 71 out of 150 patients (47%).

No other baseline hemodynamic parameters were predictors of normalization of hemodynamics.

### Improvement of pulmonary hemodynamics

One hundred and nine patients (73%) had preoperative PVR >500 dyn.sec.cm^-5^. Seventy of these 109 patients (64%) improved their PVR by at least 50% to reach a value of less than 500 dyn.sec.cm^-5^. When mPAP was included in the multivariate model, TL_CO_ (adjusted for FEV1/FVC ratio) was a significant predictor of hemodynamic improvement (OR, 1.06; 95% CI, 1.01 to 1.10; p = 0.009). When PVR was included in the multivariate model, TL_CO_ (adjusted for FEV1/FVC ratio) remained a significant predictor of hemodynamic improvement (OR, 1.07; 95% CI, 1.02 to 1.11; p = 0.004) (**Table 5**).

**Table 5:** Predictive factors of hemodynamic improvement*.

	Univariable Analysis		Multivariable Analysis	
	OR (95%CI)	p	OR (95%CI)	p
**Clinical Characteristics**				
**Age—years**	0.98 (0.95–1.01)	0.28	**1.00 (0.96–1.04)**	**0.95**
**Functional Characteristics**				
**NYHA functional class**	0.62 (0.32–1.20)	0.15		
**6MWD (meters)**	1.003 (1.000–1.006)	**0.023**	**1.004 (1.000–1.008)**	**0.052**
**Hemodynamic Characteristics**				
**mPAP (mmHg)**	1.10 (1.04–1.16)	<0.001		
**CO (L.min^-1^)**	0.80 (0.56–1.16)	0.24		
**PVR (dynes.s.cm^-5^)**	1.002 (1.000–1.003)	0.022	**1.005 (1.002–1.008)**	**0.001**
**Spirometric Characteristics**				
**TL_CO_ (% of predicted value)**	1.051 (1.016–1.088)	0.004	**1.066 (1.021–1.113)**	**0.004**
**FEV1/FVC (%)**	1.082 (1.025–1.143)	0.005	**1.075 (1.014–1.158)**	**0.046**
**Therapeutic Characteristics**				
**Introduction of PAH targeted treatment before PEA**	0.74 (0.27–2.00)	**0.55**		

*Abbreviations*: OR, Odds Ratio; CI, Confidence Interval; NYHA, New York Heart Association; 6MWD, 6-min walk distance; CO, cardiac output; PVR, pulmonary vascular resistances; FEV1, forced expiratory volume in one second; FVC, forced vital capacity; TL_CO_, transfer coefficient of the lung for carbon monoxide; PAH, pulmonary arterial hypertension; PEA, pulmonary endarterectomy.

* Hemodynamic improvement was defined by a postoperative decrease in PVR by at least 50% to reach a value of less than 500 dynes.s.cm^-5^ and occurred in 70 out of 109 patients (64%).

### Improvement of NYHA functional class

Postoperatively, 73% of patients (110/150 patients) improved their NYHA functional class by at least one class. In multivariate analysis, baseline TL_CO_, functional class and FEV1/FVC predicted improvement of NYHA functional class (**Table 6**). A worse NYHA functional class at baseline predicted a greater improvement in NYHA functional class after surgery.

**Table 6:** Predictive factors of functional improvement*.

	Univariable Analysis		Multivariable Analysis	
	OR (95%CI)	p	OR (95%CI)	p
**Clinical Characteristics**				
**Age—years**	0.99 (0.96–1.02)	0.45		
**Associated Medical conditions**	3.00 (0.65–13.75)	0.16	**2.42 (0.28–20.78)**	**0.42**
**Functional Characteristics**				
**NYHA functional class**	1.86 (1.000–3.466)	0.05	3.11 (1.21–8.00)	**0.019**
**6MWD (meters)**	1.001 (0.999–1.003	0.30		
**Hemodynamic Characteristics**				
**mPAP (mmHg)**	1.029 (0.996–1.064)	0.089	1.027 (0.984–1.072)	**0.22**
**CO (L.min^-1^)**	1.10 (0.84–1.45)	0.48		
**PVR (dynes.s.cm^-5^)**	1.000 (0.999–1.001)	0.91		
**Spirometric Characteristics**				
**TLCO (% of predicted value)**	1.036 (1.005–1.069)	0.024	**1.042 (1.006–1.079)**	**0.021**
**FEV1/FVC (%)**	1.041 (0.998–1.085)	0.064	**1.052 (1.002–1.104)**	**0.042**
**Therapeutic Characteristics**				
**Duration of PAH targeted treatment before PEA (months)**	0.93 (0.84–1.02)	0.13	**0.898 (0.802–1.006)**	**0.65**

*Abbreviations*: OR, Odds Ratio; CI, Confidence Interval; NYHA, New York Heart Association; 6MWD, 6-min walk distance; CO, cardiac output; PVR, pulmonary vascular resistances; FEV1, forced expiratory volume in one second; FVC, forced vital capacity; TL_CO_, transfer coefficient of the lung for carbon monoxide; PAH, pulmonary arterial hypertension; PEA, pulmonary endarterectomy.

* Functional improvement was defined by improvement of at least one NYHA functional class and occurred in 110 out of 150 patients (73%).

## Discussion

The present study assessed the factors influencing short- and long-term mortality, the predictors of functional improvement, and the factors predicting improvement or normalization of pulmonary hemodynamics in patients who underwent PEA for CTEPH.

### Short- and long-term mortality

In our cohort, the mortality rates at 1 month, 1 year and 3 years were 2.9%, 6.9% and 7.5%, respectively, which are similar to other CTEPH centres around the world [4, 8, 12–15]. Our study supports the importance of preoperative PVR in predicting early post-operative, 1- and 3-year mortality and of age in predicting 1- and 3-year mortality. The main causes of deaths observed during the early postoperative period were related to right ventricular failure. In contrast, long-term mortality was associated with other causes than CTEPH.

Several studies have shown that high preoperative PVR was associated with an increase in early postoperative mortality [6, 9, 11, 15]. Tscholl *et al*. were first to demonstrate that patients with a high preoperative PVR (greater than 1136 dyn.sec.cm^-5^) had a markedly higher mortality risk [11]. Dartevelle *et al*. also reported a prognostic influence of PVR, with in-hospital mortality increasing when preoperative PVR was higher than 900 dyn.sec.cm^-5^[9]. Lastly, in a large series of CTEPH patients treated by PEA at the University of California–San Diego, Madani *et al*. showed that preoperative PVR levels correlated with perioperative mortality, with patients experiencing a higher mortality when PVR exceeded 1000 dyn.sec.cm^-5^[6]. Interestingly, in the present study, preoperative PVR was also identified as an independent risk factor of late death (at 1 and 3 years) in multivariate analysis, while in other studies, PVR measured immediately after PEA[6, 7, 10] or at 3- to 12-month post-PEA was found to be a predictor of long-term mortality [8, 12]. Our study confirms that the most important risk factor for mortality remains the preoperative hemodynamic impairment assessed by the level of PVR. We found a threshold of 800 dyn.sec.cm^-5^ for identification of patients at high or low risk of death with sensitivity and specificity of 77% and 60%, respectively. In addition, patient selection for surgery is a complex process that is based on the concordance between the degree of obstruction and the level of PVR. Therefore, our cut-off value of PVR must be interpreted cautiously and should not be considered as the only criteria for surgical treatment decision. Lastly, age was also a predictor of 1 and 3-year mortality but did not predict in-hospital mortality. These results confirm that an advanced age is not systematically a criterion for inoperability [19, 20].

We observed a trend towards a deleterious effect of the use of PAH targeted therapy before PEA with mortality increasing by 50% per month of use. In our study, PAH-targeted medical therapy was more often administrated in patients with more severe hemodynamic derangements. Preoperative medical therapy to reduce PVR before PEA has been explored in operable patients with severe pulmonary haemodynamic impairment. Indeed, some studies have shown that preoperative PAH targeted therapy improved hemodynamics before surgery and postoperative outcomes [21, 22]. In a retrospective review of patients referred to the University of California at San Diego for PEA between 2005 and 2007, Jensen *et al*. observed that the use of medical therapy before PEA had increased from 19.9% in 2005 to 37% in 2007. Their analysis revealed that the use of PAH targeted treatment before surgery resulted in delayed referral for PEA, was not associated with improvement in postoperative pulmonary hemodynamics and had no impact on postoperative outcome [23]. More recently, an international prospective registry including 27 centres in Europe and Canada assessed the long-term outcomes of a cohort of operated and not-operated patients. The authors found that 29% of operated patients had received bridging therapy with PAH-targeted drugs before PEA. These patients had higher preoperative PVR in comparison with those who did not receive bridging therapy but postoperative PVR and the rate of PEA related complications did not differ between the 2 groups. In addition, one of the independent predictors of mortality, discovered in the multivariate analysis, was bridging therapy with PH drugs [14]. Thus, these observations reinforce the idea that PAH targeted therapy should not delay a patient’s referral to expert centres for PEA and if indicated, should be started after a multidisciplinary decision.

### TL_CO_ as a predictive factor of postoperative improvement

In the present study, we demonstrated for the first time that preoperative TL_CO_ predicted improvements in hemodynamics and NYHA functional class after PEA. Despite these results, we did not find that TL_CO_ was associated with postoperative mortality.

Previous studies have shown that TL_CO_ was a predictive factor of short- and long-term mortality. Condliffe *et al*. assessed factors influencing mortality in a cohort of 236 patients who underwent PEA and reported that preoperative TL_CO_ was an independent predictor of perioperative mortality [24]. More recently, Suda *et al*. investigated the relationship between TL_CO_/alveolar ventilation (TL_CO_/V_A_) and long-term outcomes after PEA and found that decreased TL_CO_/V_A_ was associated with poor long-term outcomes. In addition, the authors showed the absence of correlation between TL_CO_/V_A_ and proximal obstruction suggesting that TL_CO_/V_A_ might be related to distal or microvascular disease [25]. In another study, Zoia *et al*. have reported that patients who underwent successful PEA improved gradually their TL_CO_ after surgery due to the reversal of microvascular disease [26]. Thus, TL_CO_ might reflect the degree/severity of small vessel vasculopathy and could be a simple tool to predict postoperative improvement.

The relationship between the decrease in TL_CO_ and microvascular disease remains poorly understood [27]. Bernstein *et al*. showed that TL_CO_ was slightly reduced in CTEPH patients. They demonstrated that this decrease in TL_CO_ was predominantly caused by a reduction in pulmonary membrane diffusion capacity (Dm) and to a lesser extent by a low pulmonary capillary blood volume (Vc) [28]. Steenhuis *et al*. noted that Dm was also strongly associated with PVR. They hypothesized that the reduction in Dm was due to microvascular disease and, more specifically, to an alveolocapillary membrane thickening [29]. Interestingly, Dorfmüller *et al*. described histological findings from 17 CTEPH patients who were transplanted either after failure of PEA or because of inoperable disease. They reported an involvement of small pulmonary arteries but also venous remodelling and focal capillary haemangiomatosis [30].

Whether TL_CO_ may be a predictor of mortality and a predictive factor of improvement of hemodynamics and NYHA functional class after PEA requires further confirmation in larger prospective trials.

### Limitations

We acknowledge some limitations such as the retrospective and monocentric nature of the present study. In addition, some patients were not evaluated for secondary endpoints because of lack of hemodynamic data. The low mortality rate after PEA limited the power of the multivariate analysis. Lastly, we did not analyse the impact of the degree and location of pulmonary vascular obstruction determined by CT scan and pulmonary angiography on the post-PEA outcome because no objective radiological score has been yet established in CTEPH. Moreover, the surgical classification schema to separate patients between proximal (main and lobar) vs distal (segmental and subsegmental) chronic thromboembolic disease resection was not available for some patients. It is possible that this distinction is an important factor to predict longterm outcome after surgery. Finally, we identified different factors predicting mortality, improvement in hemodynamics, normalization of hemodynamics and improvement in functional status. These findings may be due to a lack of statistical power.

In conclusion, PEA is associated with an excellent long-term survival and a marked improvement in clinical status and hemodynamics. Preoperative PVR is a major determinant of short- and long-term mortality whereas TL_CO_ may predict postoperative improvement of hemodynamics and NYHA functional class. These findings need to be confirmed by prospective studies including large cohorts of CTEPH patients.

## Supporting information

S1 Appendix Causes of deaths. (DOCX)
