# Supplementary material for: Factors predicting outcome after pulmonary endarterectomy
Source: PLoS One. 2018 Jun 21;13(6):e0198198. doi: 10.1371/journal.pone.0198198 (PMC6013172; doi:10.1371/journal.pone.0198198)
Supplement: S1 Appendix — (DOCX) [file pone.0198198.s001.docx]

**Annexe : Causes of Deaths**

| Time after PEA | Mortality – n (%) | Causes of death |
| --- | --- | --- |
| 1 month | 5 (2.9%) | -3 right ventricular failure  -2 septic shock caused by pneumonia |
| 6 months | 8 (4.6%) | -2 right ventricular failure  -1 unknown cause |
| 1 year | 12 (6.9%) | -1 acute pancreatitis  -2 unknown causes  -1 sudden death |
| 3 years | 13 (7.5%) | -1 leukemia |
| > 3 years | 19 (11%) | -2 right ventricular failure  -1 acute renal failure  -1 cerebral lymphoma  -1 acute pancreatitis |

*Abbreviations*: PEA, pulmonary endarterectomy.
